# Belowground biomass of alpine shrublands across the northeast Tibetan Plateau

**DOI:** 10.1002/ece3.6275

**Published:** 2020-04-19

**Authors:** Nie Xiuqing, Dong Wang, Yang Lucun, Fan Li, Zhou Guoying

**Affiliations:** ^1^ Key Laboratory of Tree Breeding and Cultivation of the State Forestry Administration Research Institute of Forestry Chinese Academy of Forestry Beijing China; ^2^ Key Laboratory of Tibetan Medicine Research Northwest Institute of Plateau Biology Chinese Academy of Science Xining China; ^3^ Research Institute of nature protected Area Chinese Academy of Forestry Beijing China; ^4^ Qinghai Key Laboratory of Qing‐Tibet Biological Resources Xining China; ^5^ Institute of Qinghai Meteorological Science Research Xining China

**Keywords:** alpine shrublands, belowground biomass, mean annual precipitation, mean annual temperature, Tibetan Plateau

## Abstract

Although belowground biomass (BGB) plays an important role in global cycling, the storage of BGB and climatic effects on it are remaining unclear. With data from 49 sites, we aimed to investigate BGB and its climatic controls in alpine shrublands in the Tibetan Plateau. Our study showed that the BGB (both grass‐layer and shrub‐layer biomass) storage in the alpine shrublands was 67.24 Tg, and the mean BGB density and shrublands area were 1,567.38 g/m^2^ and 4.29 × 10^4^ km^2^, respectively. Shrub layer had a larger BGB stock and accounted for 66% of total BGB this area, while only 34% was accumulated in the grass layer. BGB of the grass layer in the Tibetan Plateau shrublands was larger than that of Tibetan alpine grasslands, indicating that shrubland ecosystem played a critical importance role in carbon cycle on the Tibetan Plateau. The BGB in the grass layer and shrub layer demonstrated different correlations with climatic factors. Specifically, the effects from mean annual temperature on shrub‐layer BGB were not significant, similarly to the relationship between mean annual precipitation and grass‐layer BGB. But shrub‐layer BGB had a significantly positive relationship with mean annual precipitation (*p* < .05), while grass‐layer BGB showed a trend of decrease with increasing mean annual temperature (*p* < .05). Consequently, the actual and potential increases of BGB varied due to different increases of mean annual precipitation and temperature among different areas of the Tibetan Plateau. Therefore, in the warmer and wetter scenario, due to contrary relationships from mean annual precipitation and temperature on shrub‐layer BGB and grass‐layer BGB, it is necessary to conduct a long‐term monitoring about dynamic changes to increase the precision of assessment of BGB carbon sequestration in the Tibetan Plateau alpine shrublands.

## Graphical abstract

The BGB storage (both shrub layer biomass and grass layer biomass) in the alpine shrublands as 67.24 ± 26.34 Tg. The BGB in grasslands layer and shrublands layer have different responses to changing climatic factors.

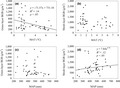

## 1 INTRODUCTION

The plants biomass is the organic matter, which obtains from photosynthetic activity. The biomass not only can provide significant information about carbon (C) cycle but also has a significant role in the global carbon cycle (Liu, Chen, Song, & Han, 2015b; Scurlock, Johnson, & Olson, 2002). Many researchers have focused on aboveground biomass (AGB). Belowground biomass (BGB) has attracted less attention, especially on the Tibetan Plateau. The storage of AGB has been estimated for both grasslands and shrublands of the Tibetan Plateau (Nie et al., 2018; Yang, Fang, Pan, & Ji, 2009), but BGB storage has not been measured to the same degree. To make up for this shortage, many studies have focused on BGB in shrublands (Cui, Zhang, Shen, Liu, & Wang, 2017; Jin, Li, Wang, Zhang, & Lei, 2012; Yang et al., 2017). However, few studies have paid attention to BGB in the Tibetan Plateau shrublands (Jin et al., 2012; Yang et al., 2017). Although BGB was less focused on in Tibetan vegetation, BGB may also play significant role in carbon (C) cycling. It has been demonstrated that the root biomass: Shoot biomass (R/S) was 5.8 in the alpine grasslands on the Tibetan Plateau (Yang, Fang, Ji, & Han, 2009), which meant that compared with AGB, more C storage stored in BGB in grasslands. In the Tibetan Plateau alpine shrubland, the R/S was 0.95 for woody plants, which meant that BGB also played significant roles in C storage (Nie, Yang, Yang, & Zhou, 2016). However, researches about BGB in the Tibetan Plateau shrubs are existed shortages. Some have only estimated BGB in shrub layer without involving BGB in grass layer (Jin et al., 2012; Nie et al., 2016) or have only considered BGB at the species level (*Sophora moorcroftiana*) (Cui et al., 2017). Thus, a comprehensive survey was needed to estimate BGB on the Tibetan Plateau, taking into account grass‐layer BGB and shrub‐layer BGB.

Shrubs are an important component of ecosystems at high latitudes and in alpine areas and also have a significant role in ecosystem functions (Liu, Chen, Song, & Han, 2015a; Wang, Zhou, Yang, & Li, 2003). Shrublands cover 19% of total areas on the Tibetan Plateau (Hu, Wang, Liu, & Fu, 2006). Plants have higher efficiency of carbon uptake at high altitude region, with higher transpiration rate and stomatal conductance (Kumar, Kumar, & Ahuja, 2005) and plants form higher altitudes having a greater rate of photosynthesis (Hovenden & Brodribb, 2000). Using methods of repeated photography, long‐term ecological monitoring, and dendrochronology, researchers have found shrubland expansion in the cold areas, which has resulted into significant biomass changes during the recent decades (Myers‐Smith, 2011). The recent changes of climate, including increased temperature, have been demonstrated that made a contribution to a rapid increase in shrublands in China (Fang, Guo, Piao, & Chen, 2007). Meanwhile, Piao et al. (2009) reported that information about shrubland ecosystems cabron was significant scarce and the shrubland ecosystems’ contribution to the China carbon sink was unknown. Therefore, it is an urgent necessary to increase the precision of BGB evaluations to provide significant information of carbon in alpine shrubland ecosystems (Piao et al., 2009).

Most studies investigating the relationships between biomass and climatic factors have focused on AGB for decades (Epstein, Lauenroth, & Burke, 1997; Fang et al., 2005; Nie et al., 2018; Sala, Parton, Joyce, & Lauenroth, 1988; Yang, Fang, Ji, et al., 2009). It has been demonstrated that precipitation could significantly shape AGB in grasslands ecosystem and based on the understanding informs predictions of production biomass changes (Fang et al., 2005; Jobbágy, Sala, & Paruelo, 2002; Ni, 2004). In Tibetan Plateau grasslands, temperature was found to only play a minor role in shaping AGB (Yang, Fang, Pan, et al., 2009), which was different from the areas of arctic and alpine environments, where temperature had larger effects on the growth of tundra plants than precipitation (Doak & Morris, 2010; Klanderud, 2005). However, BGB, including grass‐layer BGB and shrub‐layer BGB, has not been studied in Tibetan shrublands. Plants generally allocate more biomass to limited organs for acquiring the lacking resources (McCarthy & Enquist, 2008). Climatic factors (temperature and precipitation) are necessary and controlling condition for plant growth (Mokany, Raison, & Prokushkin, 2006; Wynn et al., 2006). Therefore, temperature and precipitation play a significant role in determining plant biomass allocation between AGB and BGB across ecosystem.

Climatic changes on the Tibetan Plateau have been notable in recent years (Guo et al., 2015). Specifically, the increasing mean annual temperature (MAT) on the Tibetan Plateau was 0.05°C, and mean annual precipitation (MAP) also shows an increasing trend, with a rate of increase of 1.02 mm (Yang, 2018). Climatic changes have a significant role in shaping biomass allocation between AGB and BGB in terrestrial biomes (Mokany et al., 2006), which contributes to predict change of biomass. Thus, climatic factors may inescapably affect the BGB in the Tibetan Plateau alpine shrublands. Therefore, a quantitative understanding of how the grass‐layer BGB and shrub‐layer BGB on the plateau are affected from climatic factors is necessary in order to the precisely evaluate about carbon dynamics resulted from climatic change. The amount of C applied/supplied in the form of cattle or sheep dung is not only important in determining BGB, but also can regulate ecosystem services (Isbell et al., 2011) and identify land degradation processes (Maestre & Escudero, 2009). Due to lack of these data, relevant analyze and discuss data were not involved in this study. We surveyed BGB at 49 sites in shrubland ecosystems from 2011 to 2013, with the aim of answering the following two questions: (a) How much BGB is stored in the Tibetan Plateau alpine shrubland ecosystems? (b) How have shrub‐layer BGB and grass‐layer BGB responded on changes of MAT and MAP?

## 2 MATERIALS AND METHODS

### 2.1 Study area

**Figure 1:**
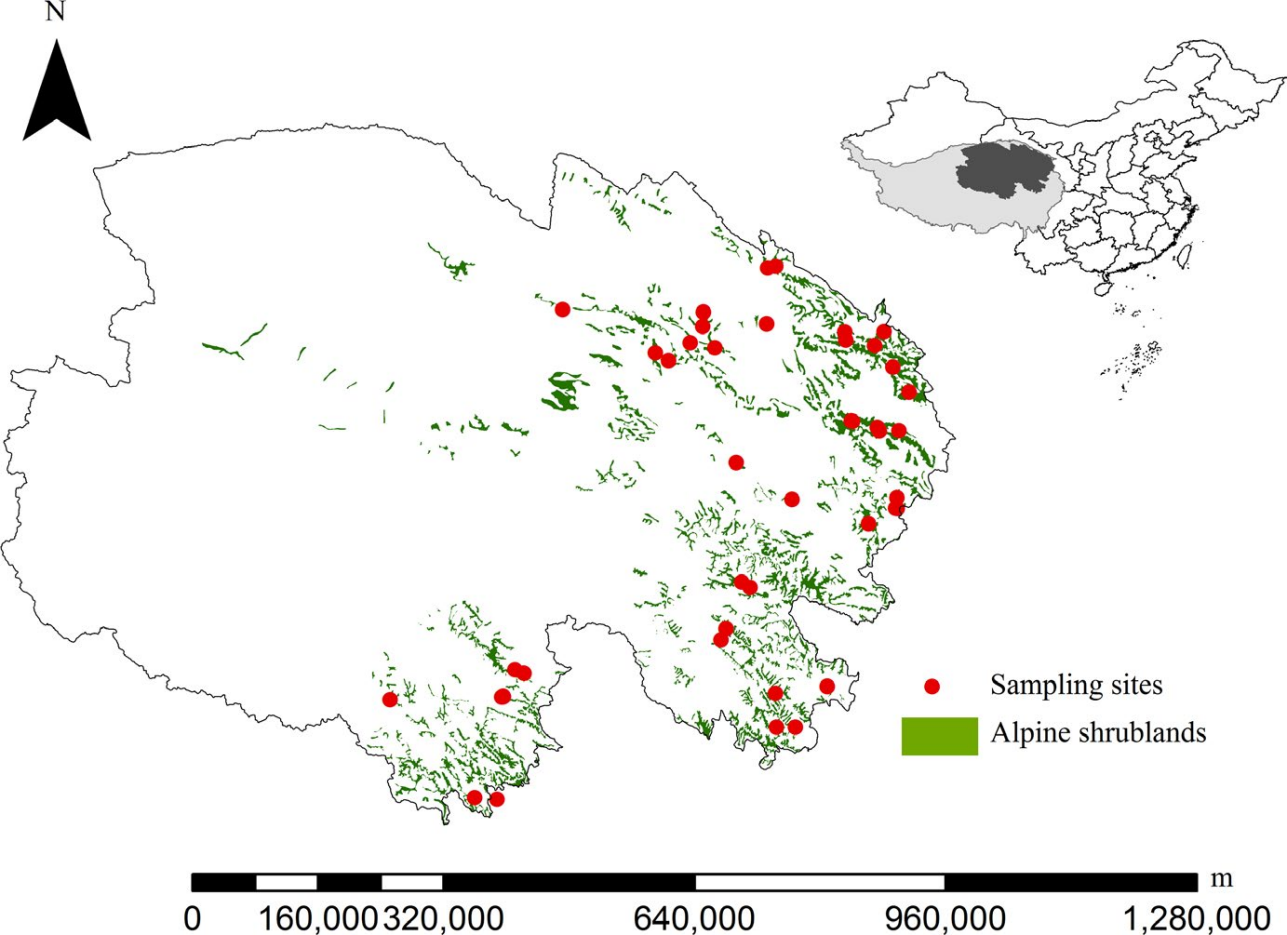
Distribution of sampling sites in the shrublands of the Tibetan Plateau, and the vegetation map was based on China's vegetation atlas 1:1,000,000(Chinese Academy of Science, 2001)

The study regions are situated on the northeast Tibetan Plateau (Figure 1), which is the highest and largest plateau on Earth and can be regarded as a “third pole” with an mean altitude of more than 4,000 m (Yang et al., 2008). The study region extends from longitude 95.30° to 102.38° E and latitudes 31.88° to 38.06° N. The dominant shrubs are as following, for example, *Rhododendron capitatum*, *R. thymifolium*, *Potentilla fruticosa,* and *Sibiraea laevigata*, and the representative herbs are mainly *Poa pratensis*, *Elymus nutans*, and *Stipa purpurea* (Zhou, Wang, & Du, 1987) . According to global world data of the Harmonized World Soil Database (HWSD), the main soil types are classified as Gelic Leptosols, Eutric Leptosols, and Molic Leptosols. The MAT and MAP of the study regions were −5.6–8.9°C and 17.6–764.4 mm, respectively (Zhang, 2009).

### 2.2 Sampling of BGB and laboratory measurement

A total of 147 plots were collected across 49 sites (i.e., each site including three plots) in the Tibetan Plateau shrublands during the period of July and August from 2011 to 2013. The detail information of each site has been provided in the Table S1. The distance was between 5 m and 50 m between any two plots. Each plot of 5 m × 5 m was used for ecological investigation, such as definite dominant species. According to the sampling method for shrubland ecosystem, the sampling size should be not smaller than 1m × 1 m (Technical Manual Writing Group of Ecosystem Carbon Sequestration Project, 2015). Due to the limited manpower and financial resources, in the each plot, one subplot of 1 m × 1 m was sampled, and the total of all plants in the subplots was dug up to determine BGB. Generally, we dug a pit of 1 m depth, or we dug until the parent material horizon to collect root samples. The BGB comprised biomass including the grass layer and the shrub layer. Grass and shrub roots grow together in each plot, and based on lignify of BGB, we separated them in each plot. The criteria of distinguishing grass and shrub root were whether they are herbs or woody. Specifically, the shrub layer primarily consisted of woody biomass, while grass layer composed by herbs biomass. Meanwhile, combining with AGB, we can easily distinguish grass‐layer BGB and shrub‐layer BGB. Then, we only retained root sample and cleaned up the other parts (Technical Manual Writing Group Of Ecosystem Carbon Sequestration Project, 2015). The biomass samples were all weighed to the nearest 0.01 g after it was oven‐dried to a constant mass at 65°C. Although the amount of C applied/supplied in the form of cattle or sheep dung can affect soil nutrient (Du, Cai, Wang, Zhang, & Du, 2019), and consequently, can affect BGB. Due to limitations of resources, the responding research was not conducted.

### 2.3 Climate data

In order to evaluate the potential effects from climatic variables on BGB, we extracted every sampling climate factors from China Integrated Meteorological Information Service System (http://www.data.cma.cn). The MAP and MAT were compiled using climatic data on the Tibetan Plateau from 2010 to 2011. Based on original climatic data, we obtained two rasters of MAP and MAT by kriging method in ArcGIS 10.2 (http://www.esri.com/). The method of extract values to points in spatially analyst tool was employed to interpolated climatic data of each site according to longitude and latitude. Those data were spatially interpolated from 50 climate stations, and these stations are located a mean altitude of more than 3,000 m across the Tibetan Plateau (Nie et al., 2019).

### 2.4 Data analysis

To estimate BGB storage on the Tibetan Plateau, we calculated BGB storage in the grass layer and shrub layer, respectively, according to the equation (1) (Fang et al., 2014).

(1) M=A×D

where *M* is storage of BGB (Tg), *A* represents the shrubland area (10^4 ^km^2^) and *D* represents the biomass density (kg/m^2^). Data of shrubland area were acquired using China's vegetation map with a scale of 1:1,000,000 (Chinese Academy of Science, 2001).

The SPSS 22.0 (SPSS Inc.) was used to conduct statistical analysis. Regression analyses were performed to estimate the existed significant relationship (*p* < .05) between the climatic factors and BGB using curve measurement. Figures were plotted by SigmaPlot 12.5 (Systat software, Inc, Point Richmond). The result from one‐way analysis of variance showed that soil type has no significant effects on grass‐layer BGB (Figure S1a), and shrub‐layer BGB (Figure S1b). And due to the insignificant relationship, we did not expand discussion.

## 3 RESULTS

### 3.1 Sizes and storage of BGB

**Figure 2:**
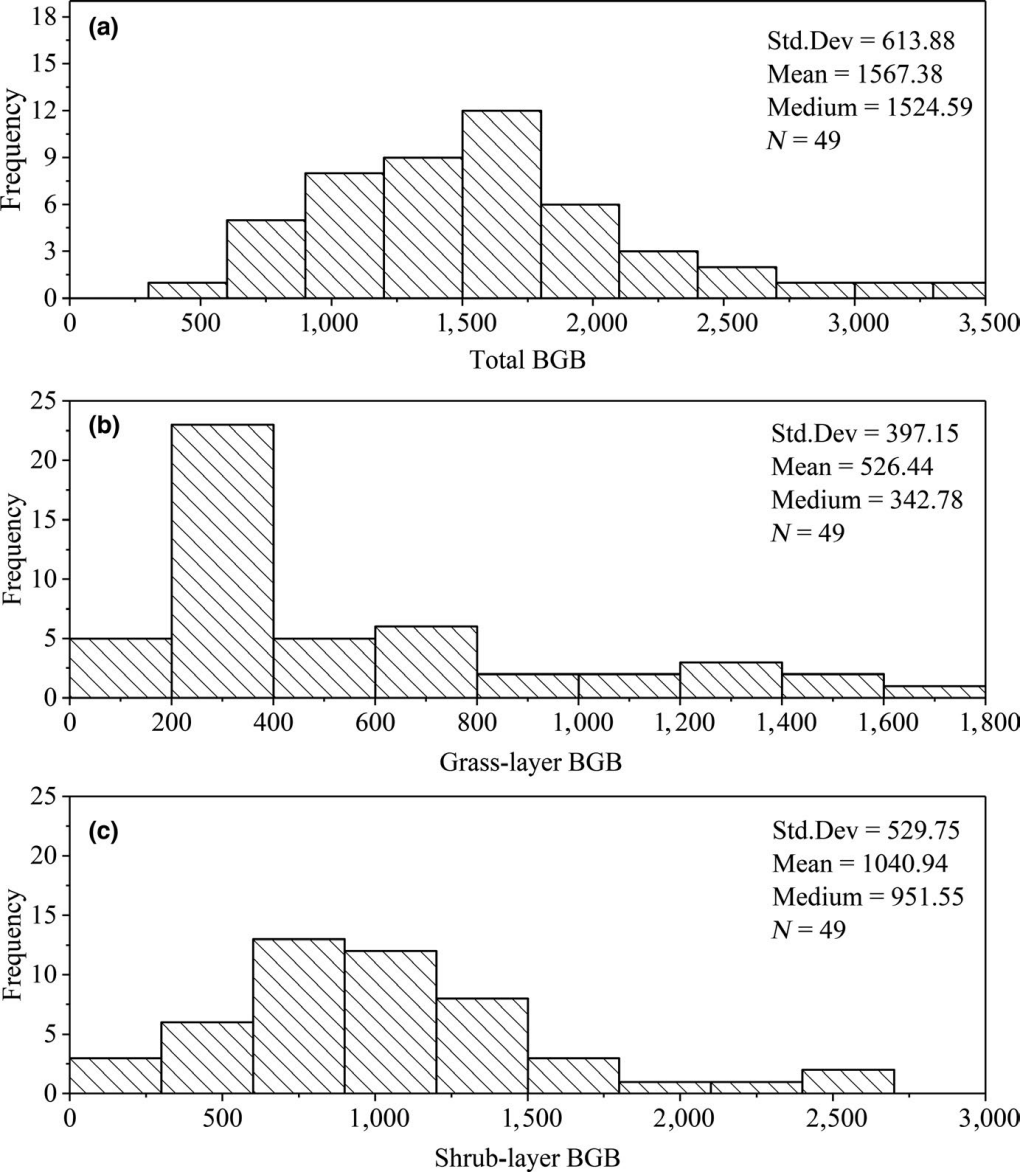
Frequency distributions of (a) total belowground biomass (BGB), (b) grass‐layer BGB, and (c) shrub‐layer BGB. Mean values, medium values, and standard deviation are presented

**Figure 3:**
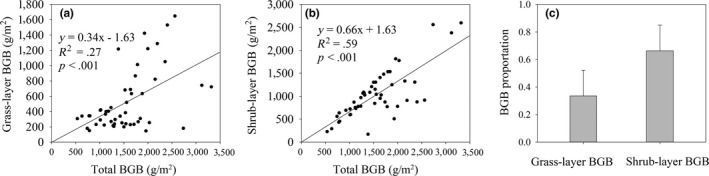
Relationship and proportion between grass‐layer belowground biomass (BGB), shrub‐layer BGB, and total BGB

Shrub‐layer BGB, grass‐layer, and total BGB exhibited large variations across the Tibetan Plateau shrublands, ranging from 170.21 to 2,597.28 g/m^2^, 145.68 to 1,648.15 g/m^2^, and 533.85 to 3,319.48 g/m^2^, respectively (Table 1). The median values of grass‐layer BGB, shrub‐layer BGB, and total BGB were 342.78 g/m^2^, 951.55 g/m^2^, and 1,524.59 g/m^2^, respectively (Figure 2). The overall mean BGB was 1,567.38 g/m^2^ in alpine shrubland ecosystems, specifically, a greater biomass density in the shrub layer of 1,040 0.94 g/m^2^ than that in the grass layer of 526.44 g/m^2^ (Figure 2). Total BGB storage was 67.24 ± 22.73 Tg (Table 1), mainly stored in the shrub layer, and accounted for 66% of BGB, with the only 34% accounting in grass layer (Figure 3).

**Table 1:** Storage of belowground biomass (BGB) in alpine shrubland ecosystems of the Tibetan Plateau

Vegetation type	Area (10^4^ km^2^)	BGB density (g/m^2^)		BGB storage (Tg)
		Mean	Range	
Shrub layer	4.29	1,040.94	170.21–2597.28	44.66 ± 22.73
Grass layer	4.29	526.44	145.68–1648.15	22.58 ± 17.04
Total	4.29	1,567.38	533.85–3319.48	67.24 ± 26.34

### 3.2 Effects of MAP and MAT on BGB

**Figure 4:**
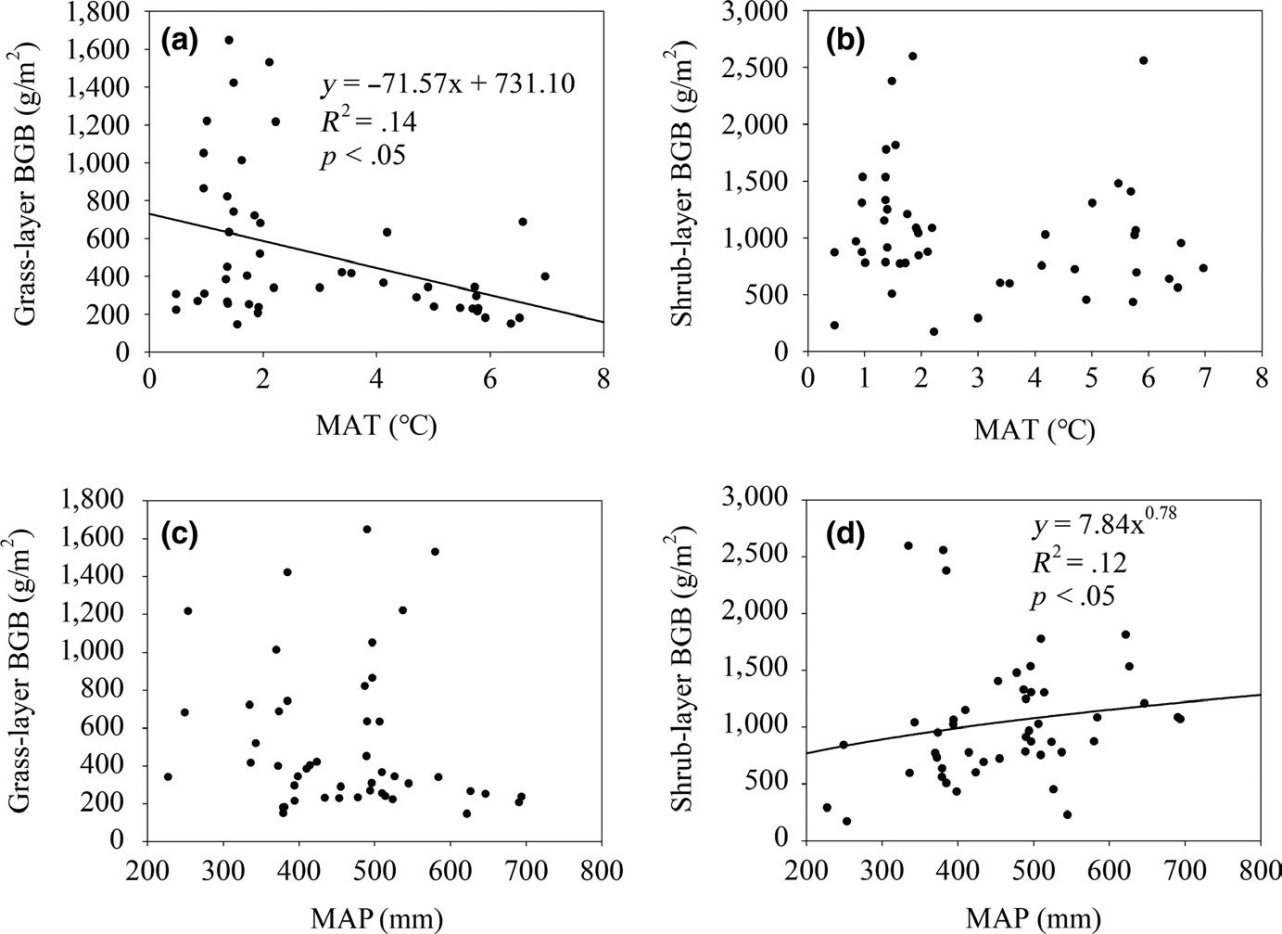
Regressions of grass‐layer belowground biomass (BGB) and shrub‐layer BGB on mean annual temperature (MAT) (a, b), and mean annual precipitation (MAP) (c,d)

Overall, climatic factors have had different effects on grass‐layer and shrub‐layer BGB. Specifically, the grass‐layer BGB decreased with increasing MAT (*p* < .05) (Figure 4a), but MAT did not have significant effects on shrub‐layer BGB (Figure 4b). Although MAP did not have significant effects on grass‐layer BGB (Figure 4c), shrub‐layer BGB showed a trend of increase with increasing MAP (*p* < .05) (Figure 4d).

## 4 DISCUSSION

### 4.1 Sizes of BGB

The average BGB of the shrub layer in the Tibetan Plateau shrublands was 1,040.94 g/m^2^, which is smaller than that in the northern regions of China's alpine shrublands (1,320 g/m^2^) (Yang et al., 2017). The Tibetan Plateau climate is cold and arid across the main body of the plateau (Ding et al., 2016). Compared with northern areas of China, the dry and cold environment may limit the growth of plants, resulting in the smaller BGB in the shrub layer on the plateau.

Yang, Fang, Pan, et al. (2009) have calculated that alpine grassland BGB was 330.5 g/m^2^. We found that the BGB of the grass layer on the Tibetan Plateau was 526.44 g/m^2^. There are two reasons to explain the difference. Firstly, for the herbs in shrublands ecosystem, the growth temperature may be higher than that in grasslands ecosystem. Researcher has demonstrated that the existing of shrubland canopies has great effects on permafrost soil temperature (Myers‐Smith et al., 2011) and can result in raising the soil temperature (Sturm et al. 2001). Thus, it may contribute to the growth of grass under shrubland canopy, and consequently shapes a larger grass‐layer BGB. Secondly, the shrubland canopy can also make a contribution to nitrogen cycling, more litter input and litter decomposition (Myers‐Smith, 2011), which makes shrubland contribute to promote the growth of plants of grass layer. Hence, grass‐layer BGB in the shrublands of the Tibetan Plateau is larger than that in the alpine grasslands on the Tibetan Plateau. It has been demonstrated a similar result for AGB, specifically, the grass‐layer AGB in alpine shrublands also larger than that of Tibetan Plateau alpine grasslands (Nie et al., 2018).

### 4.2 Relationship between temperature and BGB

The shrub‐layer BGB did not show any significant relationship with the MAT gradient on the Tibetan Plateau. Similarly, BGB of alpine grasslands also showed no any significant relationship along changing MAT (Yang, Fang, Ma, Guo, & Mohammat, 2010), which indicates that these two vegetation types on the Tibetan Plateau may have similar responses to the MAT. It be can inferred that the BGB in both alpine grasslands and shrublands may be stable with increasing temperature. However, the BGB of the grass layer decreased with increasing temperature. Due to a shorter growing season and decreasing CO_2_ partial pressure with increasing altitude (Körner, 2007; Woodward & Pigott, 1975), plants may invest more in AGB to obtaining a higher photosynthetic rate (Nie et al., 2016), which reduce investment in BGB, and may partly explain the decrease in BGB in the grass layer of the Tibetan Plateau shrublands.

Temperature has been demonstrated that plays an important role in shaping AGB, which increased with temperature in alpine shrublands on the Tibetan Plateau (Nie et al., 2018). However, temperature only had minor effects on the BGB of the shrub layer in the Tibetan Plateau shrubland ecosystems, which suggests that in the global warming scenario plants attends to allocate more biomass to shrub‐layer AGB, which may potentially make a contribution to the expansion of shrublands.

### 4.3 Relationship between precipitation and BGB

The BGB of the shrub layer in alpine shrubland ecosystems showed an increasing trend with increasing MAP, which demonstrates that precipitation plays a significant role in determining the sizes of shrub‐layer BGB. Whereas the relationship between grass‐layer BGB and MAP was not significant, indicating that BGB responses to precipitation differed in the grass layer and shrub layer. Studies have shown that precipitation has a positive effect on BGB in the Tibetan grasslands (Yang, Fang, Ji, et al., 2009). Generally, in the areas of water shortage, precipitation makes contributions to plant growth (Callesen et al., 2003; Wynn et al., 2006). And on the Tibetan Plateau, arid was one of the most significant climatic characteristics (Ding et al., 2016; Nie, Xiong, Yang, Li, & Zhou, 2017). Hence, increasing precipitation can stimulate BGB accumulation in the shrublands on the plateau. However, precipitation only has minor effects on grass‐layer BGB in shrubland ecosystems. Owens (2006) has indicated that 35% of precipitation is intercepted by the tree canopy (Owens, Lyons, & Alejandro, 2006), which can significantly reduce the total precipitation on the grass layer in shrubland ecosystems. Thus, under a narrow range of precipitation, MAP only has minor effects on grass‐layer BGB.

Although shrublands ecosystems are considered as a huge carbon sink, evidences of information in China shrublands was lacking (Piao et al., 2009). The positive correlation between BGB and precipitation supports that the potential trend of increasing BGB carbon is existing in the Tibetan Plateau alpine shrubland ecosystems. In the global climate change scenario, precipitation is increasing by 10.2 mm every 10 years (Yang, 2018), although increases are uneven among different regions. Specifically, compared with southern regions, there has been a greater increase in precipitation than that in northern areas on the Tibetan Plateau (Duan, Yao, Wang, Tian, & Xu, 2008). Therefore, the potential biomass increase in alpine shrublands varies because of the different increases in precipitation across diverse areas. It is necessary to conduct a long‐term monitoring about dynamic changes to increase precision evaluations about potential BGB carbon sink in the Tibetan Plateau alpine shrublands.

## 5 CONCLUSIONS

The storage and the climatic determinants of BGB in the Tibetan Plateau shrubland ecosystems were evaluated by data from 49 sites from 2011 to 2013. The BGB of the alpine shrubland ecosystem was 1,567.38 g/m^2^, 66% of which was mainly stocked in the shrub layer, while only 34% accounted in grass layer. BGB of the grass layer in shrublands ecosystem was greater than that in alpine grasslands, indicating the significant role of shrubland ecosystem in carbon cycle on the plateau. Our findings reveal that climatic conditions have different effects on BGB in the grass and shrub layer on the Tibetan Plateau. Precipitation has larger effects than temperature on shrub‐layer BGB in the Tibetan Plateau alpine shrublands, and BGB in the grass layer is more sensitive to temperature than to precipitation. Under an increasing precipitation scenario, shrublands have increasing BGB carbon potential, which is diverse in different areas due to uneven increases in precipitation. Due to contrary relationships from MAP and MAT on shrub‐layer BGB and grass‐layer BGB, it is necessary to conduct a long‐term monitoring about dynamic changes to increase the precision of evaluation about potential BGB carbon sink in the Tibetan Plateau alpine shrublands.

## CONFLICT OF INTEREST

The authors declare no competing financial interests.

## AUTHOR CONTRIBUTIONS

**Nie Xiuqing:** Data curation (equal); Investigation (equal); Writing‐original draft (equal); Writing‐review & editing (equal). **Dong Wang:** Writing‐review & editing (equal). **Yang Lucun:** Investigation (equal). **Fan Li:** Investigation (equal). **Zhou Guoying:** Conceptualization (lead); Project administration (lead); Writing‐review & editing (equal).

## Supporting information

Fig S1

## Data Availability

The grass‐lay BGB and shrub‐layer BGB data across the northeast Tibetan Plateau shrublands in Dryad: https://doi.org/10.5061/dryad.kh189322v
